# Monitoring Phosphoinositide Fluxes and Effectors During Leukocyte Chemotaxis and Phagocytosis

**DOI:** 10.3389/fcell.2021.626136

**Published:** 2021-02-04

**Authors:** Fernando Montaño-Rendón, Sergio Grinstein, Glenn F. W. Walpole

**Affiliations:** ^1^Program in Cell Biology, Hospital for Sick Children, Toronto, ON, Canada; ^2^Institute of Medical Sciences, University of Toronto, Toronto, ON, Canada; ^3^Department of Biochemistry, University of Toronto, Toronto, ON, Canada; ^4^Keenan Research Centre for Biomedical Science, St. Michael's Hospital, Toronto, ON, Canada

**Keywords:** phosphoinositides, inositol lipids, macrophage, neutrophil, chemotaxis, phagocytosis, lipid biosensors, lipid signaling

## Abstract

The dynamic re-organization of cellular membranes in response to extracellular stimuli is fundamental to the cell physiology of myeloid and lymphoid cells of the immune system. In addition to maintaining cellular homeostatic functions, remodeling of the plasmalemma and endomembranes endow leukocytes with the potential to relay extracellular signals across their biological membranes to promote rolling adhesion and diapedesis, migration into the tissue parenchyma, and to ingest foreign particles and effete cells. Phosphoinositides, signaling lipids that control the interface of biological membranes with the external environment, are pivotal to this wealth of functions. Here, we highlight the complex metabolic transitions that occur to phosphoinositides during several stages of the leukocyte lifecycle, namely diapedesis, migration, and phagocytosis. We describe classical and recently developed tools that have aided our understanding of these complex lipids. Finally, major downstream effectors of inositides are highlighted including the cytoskeleton, emphasizing the importance of these rare lipids in immunity and disease.

## Introduction

Chemotaxis and phagocytosis are fundamental processes employed by myeloid cells of the immune system to protect the body from harmful invading microorganisms and maintain tissue homeostasis. Neutrophils, which are prototypical of myeloid cells, are the dominant circulating leukocytes; every day billions of neutrophils enter and exit the circulation (Teng et al., 2017). Their importance is revealed in cases of neutropenia –a decrease in the number or quality of circulating neutrophils—which results in recurrent bacterial infections (Leliefeld et al., 2016).

When pathogens break through the epithelial barriers of the host, circulating neutrophils are rapidly recruited to the site of infection. Upon invasion, pathogens cause the local release of molecules such as formyl peptides, peptidoglycans or lipoproteins. Further, proximal tissues are flagged for recognition by the production of inflammatory mediators (Nathan, 2006). Neutrophils sense these pathogen-associated molecules and inflammatory signals through various receptors including Toll-like receptors (TLRs) and G protein-coupled receptors (GPCRs). Upon receptor activation, neutrophils undertake diapedesis to exit blood vessels and migrate toward the site of infection within the tissue parenchyma to deploy antimicrobial functions, including but not limited to phagocytosis (Mayadas et al., 2014). They generate reactive oxygen species, release antimicrobial peptides and other cytotoxic granule components, and form neutrophil extracellular traps, all of which are effective in creating a microbicidal environment intended to eliminate pathogenic organisms (Segal, 2005). The multistep process of rolling adhesion, paracellular extravasation through endothelial junctions, migration, and ultimately the deployment of antimicrobial functions demands great morphological and functional diversity of leukocytes.

Importantly, the roles of neutrophils and other myeloid cells extend far beyond the clearance of pathogenic microorganisms. Excellent reviews are available that highlight their roles in cancer (Coffelt et al., 2016), auto-immunity (Thieblemont et al., 2016), and overall health and disease (Liew and Kubes, 2019).

Here, we describe the dynamic receptor-mediated processes of leukocyte chemotaxis and phagocytosis, two responses that are highly dependent on lipidic signals. We highlight the role that phosphoinositides, key signaling lipid molecules, play in regulating the complex series of events involved in the actin re-organization that underlies cell migration and phagocytosis. Furthermore, we describe the current tools used to study and manipulate phosphoinositides and, when possible, offer insights of their relevance to health and disease.

### Part I: Introduction to Phosphoinositides

Cellular processes, such as signal transduction, endocytosis, exocytosis, and cell migration are dependent on cellular membranes. These membranes (plasmalemmal and endomembranes) are dynamic entities that constantly undergo remodeling events, typified by fusion, budding and fission. Understandably, regulation of membrane dynamics is critical for cellular physiology. Pivotal to this regulation is the timely recruitment of effector proteins to specific membranes and to sub-domains therein. Phosphoinositides (PPIns) contribute importantly to this recruitment.

Phosphoinositides are phosphorylated derivates of phosphatidylinositol (PtdIns). They represent a minor fraction of the cellular phospholipids, yet they regulate a plethora of biological responses. PtdIns consists of a diacylglycerol (DAG) linked to D-myo-inositol-1-phosphate ring by a phosphodiester linkage. Phosphorylation can occur in the 3-, 4-, and 5-hydroxyl groups of the inositol ring, giving rise to the seven naturally occurring PPIns species. The interconversion of PPIns into other lipid species or other secondary messengers is facilitated by numerous kinases, phosphatases, and lipases which possess refined activities toward a subset of the 1-, 3-, 4-, or 5-moieities of the inositol ring. As a result, PPIns are differentially distributed among cellular membranes and within distinct membrane sub-domains, where they selectively recruit effector proteins and act as landmarks for membrane identity (Balla, 2013).

Phosphoinositides exert their functions by interacting with membrane resident molecules such as transporters and ion channels, or by selectively recruiting signaling molecules in a reversible manner. These interactions are facilitated by stereospecific inositide-binding domains present in the signaling molecules that get recruited by PPIns. The first of these domains was identified in pleckstrin (Harlan et al., 1994) and since then, the term pleckstrin homology (PH) domain has been used to refer to these homologous modules. A vast array of regulatory modules bear PH domains (Cozier et al., 2004). However, it is worth mentioning that not all PH domains bind phosphoinositides and that many also have protein-binding properties. Other classes of PPIns-binding domains have been identified: these include FERM domains that link the actin cytoskeleton to PPIns of the plasma membrane (PM) (Chishti et al., 1998), BAR and EHD domains that can sense and induce membrane curvature, and FYVE and PX domains that target several protein families to endolysosomal membranes (Chishti et al., 1998; Frost et al., 2009).

The discovery of such domains has been instrumental for studying the function and localization of PPIns (Hammond and Balla, 2015) *in situ*. As discussed below, the use of specific PPIns-binding domains as biosensors has been crucial in gaining insight of the distribution, dynamics and function of PPIns. These probes have made it possible to establish that different PPIns mark distinct membranes. Thus, PtdIns(4)P, PtdIns(4,5)P_2_, PtdIns(3,4,5)P_3_, and PtdIns(3,4)P_2_ are present almost exclusively at the PM, whereas PtdIns(4)P is recognized as the signature PPIns of the Golgi complex, and pools of PtdIns(3)P and PtdIns(4)P are present in early and late endosomes, respectively. Given the fact that many PPIns-binding proteins exhibit low affinity for their ligand, recruitment of these proteins often requires coincident detection of other binding determinants such as specific protein motifs (Simonsen et al., 1998; Wijdeven et al., 2015) or by sensing membrane curvature (Carlton et al., 2004).

### Part II: Methods to Monitor Phosphoinositides in Leukocytes

Classical biochemical techniques provided the first insight into PPIns biology in leukocytes. The discovery of PtdIns(3,4,5)P_3_ (Traynor-Kaplan et al., 1988) and PtdIns(3,4)P_2_ (Traynor-Kaplan et al., 1989), two species formed *de novo* following the stimulation of neutrophils with formylated chemotactic peptides, was possible by loading large numbers of cells with radiolabeled [^3^H]inositol or [^32^P]phosphate. Following acid extraction, inositol headgroups were deacylated by enzymatic or chemical means allowing the water-soluble radiolabelled headgroup to be isolated and analyzed. Following nuanced separation by thin-layer chromatography or by high-performance liquid chromatography (HPLC) the relative amounts of different inositide species could be inferred. Alternatively, the deacylated headgroups can be quantitatively analyzed by radioreceptor assays (Várnai and Balla, 1998), or without radiolabeling by anion-exchange HPLC coupled to conductivity detection (Nasuhoglu et al., 2002).

More recent developments in the field of lipidomics are based on ultra-high-pressure HPLC coupled to mass spectrometry (HPLC-MS/MS) (Wenk et al., 2003; Clark et al., 2011; Bui et al., 2018). Following methylation of inositol headgroups to render them electroneutral, ionization and subsequent detection allows for sensitive quantitation of the amount of different PPIns in parallel with other phospholipids. Quite importantly, although early iterations of this technique could not resolve regio-isomers (e.g., PtdIns(3,4)P_2_, PtdIns(3,5)P_2_, PtdIns(4,5)P_2_) (Kielkowska et al., 2014), harnessing differences in isomer-specific methylation patterns now allows the separation of such isomers, with the exception of PtdIns(4)P and PtdIns(5)P (Wang et al., 2016). These mass spectrometry approaches have the additional benefit of reporting fatty acyl chain length and degree of saturation, and have even been extended to analyze PPIns in whole organs (Wang et al., 2016). However, all the above biochemical readouts suffer from a major limitation: as they analyze extracts of multiple whole cells, small changes occurring asynchronously in subcellular compartments cannot be resolved. Subcellular fractionation could in principle be performed to refine the detection, but the inevitable exposure of the membranes to kinases, phosphatases and phospholipases during the lengthy fractionation schemes distorts the composition of the samples.

The sub-cellular distribution and relative levels of specific PPIns species can instead be monitored by immunostaining with specific antibodies coupled to fluorescent or chemiluminescent secondary antibodies. Originally developed by immunizing mice with immunogen-cationized inositides (Chen et al., 2002) or with liposomes containing specific PPIns (Thomas et al., 1999), this antibody collection is carried today by Echelon Biosciences. It is important to note, however, that PPIns do not contain primary amines and, therefore, cannot be easily cross-linked and stabilized during traditional fixation with paraformaldehyde. A great deal of time has been invested to develop and understand which PPIns pools can be reliably detected by immunostaining and under what conditions (Hammond et al., 2006, 2009; Yip et al., 2008). For example, preserving plasma membrane integrity requires careful adjustments to standard immunostaining methods such as the addition of the fixative glutaraldehyde, careful buffering of pH, the use of reduced temperatures and saponin for permeabilization. Unfortunately, in attempting to preserve one membrane, conditions may fail to recognize the lipid of interest in another, possibly important, cellular organelle (Hammond et al., 2009, 2012). As such, these tools should be employed only with a clear experimental focus (e.g., a defined organelle of interest in mind) and great caution. Nonetheless, immunostaining aided in revealing the presence of PtdIns(3,4)P_2_ in clathrin-coated pits (Posor et al., 2013), PtdIns(4)P in the plasma membrane (Hammond et al., 2009), and PtdIns(3,4,5)P_3_ at the leading edge of migrating leukocytes (Wang et al., 2002).

**Figure 1:**
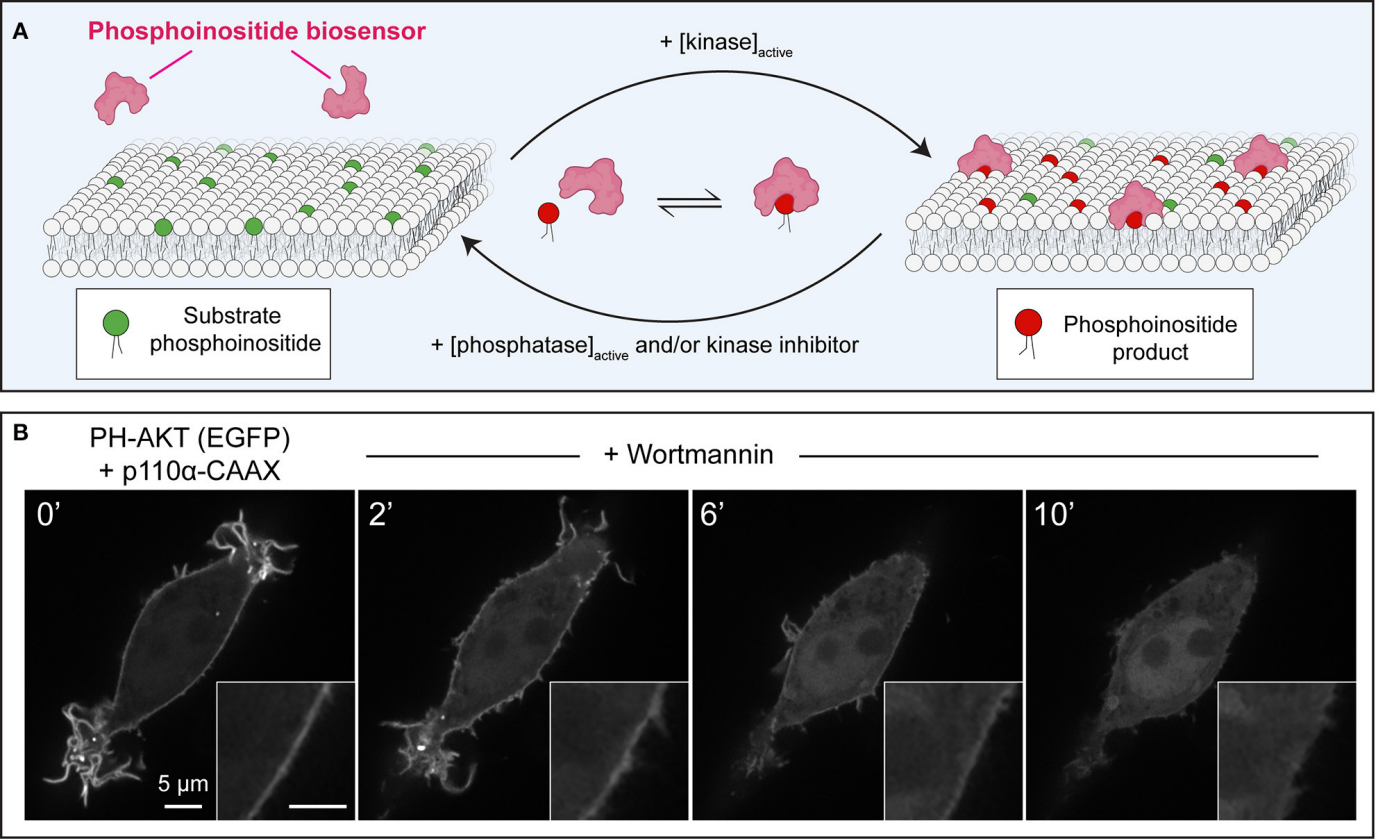
Principles of operation of phosphoinositide biosensors in leukocytes. **(A)** Model of a generic phosphoinositide-specific biosensor in equilibrium between the cytosol and membrane following activation of the kinase that generates the target lipid, or its disappearance due to phosphatase activation, or pharmacological kinase inhibition. **(B)** Dynamic redistribution of the PH-AKT biosensor in response to changes in 3-phosphorylated species. PH-AKT was co-transfected with class I PI3K-CAAX into RAW264.7 cells, leading to constitutive production of PtdIns(3,4,5)P_3_ and PtdIns(3,4)P_2_ at the plasma membrane. PI3Ks were then inhibited pharmacologically with wortmannin (100 nM). Note both the decrease in plasma membrane fluorescence and the concomitant increase in cytosolic GFP intensity over time.

The dynamic and localized responsiveness of living organisms to stimuli presents several challenges to biologists interested in PPIns signaling: the spatiotemporal nature of events, rapid turnover, and low abundance of PPIns cannot be properly appreciated by any one of the techniques discussed above. Indeed, many cellular processes necessitate the ability to track organelles or sub-domains of organelles on a second-to-second basis. The introduction of genetically-encoded biosensors based on high-affinity PPIns-binding domains provided a way to address many of these shortcomings, and led to an explosion of knowledge and interest in the field of PPIns biology (Hammond and Balla, 2015; Wills et al., 2018). PPIns biosensors exploit high-affinity, stereospecific interactions of protein domains with available lipids, an interaction that drives protein recruitment to biological membranes (Figure 1A). The PH domain of phospholipase C (PLC) δ1 was the first to demonstrate clear stereospecificity for PtdIns(4,5)P_2_ and soluble Ins(1,4,5)P_3_ (Ferguson et al., 1995; Lemmon et al., 1995). Soon after, the fusion of PH-PLCδ1 to a fluorescent protein created a reporter that has been widely utilized as an indicator of PtdIns(4,5)P_2_. The potential of GFP-tagged PH-PLCδ1 to monitor PtdIns(4,5)P_2_ in real time was demonstrated by its dynamic relocalization in response to platelet-activating factor treatment of leukocytes (Stauffer et al., 1998), and to calcium ionophore or hormone treatment of fibroblasts (Várnai and Balla, 1998). The growing knowledge of PPIns-binding domains rapidly expanded the repertoire of tools to monitor PPIns at a single-cell level (Table 1). Our toolbox today allows for monitoring of PtdIns(4,5)P_2_ (Stauffer et al., 1998; Várnai and Balla, 1998), PtdIns(4)P (Brombacher et al., 2009; Dolinsky et al., 2014; Hammond et al., 2014; Weber and Hilbi, 2014), PtdIns(3)P (Gaullier et al., 2000; Ellson et al., 2001; Kanai et al., 2001), PtdIns(3,4,5)P_3_/PtdIns(3,4)P_2_ (Frech et al., 1997; Gray et al., 1999; Watton and Downward, 1999; Manna et al., 2007), PtdIns(3,4,5)P_3_ (Klarlund et al., 1997, 2000; Venkateswarlu et al., 1998a,b; Várnai et al., 1999, 2005; Cronin et al., 2004; Manna et al., 2007), PtdIns(3,4)P_2_ (Thomas et al., 2001; Goulden et al., 2019), and PtdIns (Pemberton et al., 2020) with great selectivity, although several cautionary notes discussed below should be considered before working with these reporters.

**Table 1:** Summary of phosphoinositides, effectors in leukocytes, and biosensors used for their detection.

**Phosphoinositide**	**Effectors**	**Biosensors (Fixable)**	**Biosensor source**
PtdIns(4,5)P_2_	- WASP/N-WASP -PLC (*β, δ isoforms*) - Dynamin - AP2, Epsin, CALM (*clathrin-adaptor proteins*) - FCHo, FBP17, Amphiphysin (*BAR-domain proteins*) - Spectrin - ERM proteins - GRAF1 - PTEN	PH-PLCδ1 (+) PH-PLCδ4 (?)	Stauffer et al., 1998; Várnai and Balla, 1998; Lee et al., 2004b
PtdIns(4)P	- OSBP and ORPs - CERT - FAPP1/2 - GOLPH3 - SKIP/PLEKHM2	P4M (±) P4C (±)	Hammond et al., 2014; Weber and Hilbi, 2014
PtdIns(3,4,5)P_3_	- WAVE1/2/3 - GRP1, ARNO, Cytohesin-1 (*Cytohesin family*) - PLC (*β, γ isoforms*) - Protein Kinase B/AKT - PDK1 - BTK - SNX9/18/33 (*PX-BAR domain proteins*) - Vav1/3, Tiam1/2, P-rex1, Dock2 (*RhoGEFs*) - ARHGAP12, ARHGAP25, and SH3BP1 (*RhoGAPS*) - ARAP3, GBF1 (*ArfGEFs*) - Sos (*RasGEF*)	PH-ARNO(2G)^I303E^ (+) PH-BTK (+) PH-AKT (+)	Gray et al., 1999; Várnai et al., 1999; Watton and Downward, 1999; Goulden et al., 2019
PtdIns(3,4)P_2_	- TAPP1/2 - Protein Kinase B/AKT - SNX9/18/33 (*PX-BAR domain proteins*) - FCHSD1/2 (*F-BAR-domain proteins*) - Lamellipodin - RasGAP2* - RapGAP3*	cPH (+) PH-AKT (+)	Gray et al., 1999; Watton and Downward, 1999; Goulden et al., 2019
PtdIns(3)P	- EEA1 - Hrs (*ESCRT-0*) - WDFY2 - Rabankyrin - SNX1/2 - DFCP1, WIPI1 (*Autophagy*) - p40phox, p47phox (*NADPH Oxidase*) - PIKfyve	FYVE (+) PX (+)	Gaullier et al., 2000; Ellson et al., 2001; Kanai et al., 2001

Effectors of each phosphoinositide species are listed in **Column 2**. To provide context where possible, the relevant isoforms, protein family, and biological pathways are italicized in parentheses. Please note that protein effectors may require additional coincident signals for their recruitment to cellular membranes and/or additional co-factors in addition to the listed interacting lipid. In some instances, proteins can be recruited to sub-domains of biological membranes independently of phosphoinositides but be activated by the lipid allosterically. Effectors reported in Dictyostelium but not yet tested in mammalian leukocytes are marked with an asterisk (*). Selective lipid-binding domains are listed in **Column 3** that form the basis of phosphoinositide-specific biosensors utilized in leukocytes and other cell types. All domains are from mammalian origin except for P4M and P4C, which are derived from the Legionella pneumophila effectors SidM and SidC, respectively. The ARNO PH domain-containing biosensor specifically encodes the diglycine (2G) splice variant which exhibits strong selectivity for PtdIns(3,4,5)P_3_ over other phosphoinositide species. Additionally, interactions with host Arl GTPases are predicted to be disrupted by mutation of Isoleucine at position 303. The cPH domain-containing biosensor encodes the isolated C-terminal PH domain from TAPP1. Whether biosensor localization is retained following chemical fixation with paraformaldehyde is indicated in parentheses (+, localization retained; ±, partial disruption; ?, untested), based on the experience in our laboratory. **Column 4** provides the primary reference to the development of each biosensor, many of which have been deposited and are freely available on Addgene (https://www.addgene.org/). PH, Pleckstrin-homology domain; BTK, Bruton's Tyrosine Kinase; FYVE (Fab1, YOTB, Vac1, and EEA1); PX, Phox homology domain; PLC, phospholipase C.

Many inositide-binding domains found in nature are not selective for a single PPIns species or have too low an affinity to direct protein localization. As well, some domains exhibit protein-protein or protein-lipid interactions other than their PPIns binding and impact their localization (Hammond and Balla, 2015). Therefore, the task of generating a successful lipid biosensor is not a straightforward one. It is important and obvious that a successful biosensor must demonstrate selectivity for the lipid species of interest and depend on the lipid for its localization to the membrane. However, less obvious is the fact that the sensor should demonstrate sufficiency to recognize the lipid in a membrane where the lipid is not normally found (Hammond and Balla, 2015; Wills et al., 2018). Sufficiency for biosensor recruitment has been elucidated elegantly *in vivo* by several methods, including chemical dimerization and optogenetic activation (discussed further below) that induce ectopic synthesis of the lipid of interest in its non-native organelle. Unfortunately, several first-generation PtdIns(4)P probes did not comply with the latter requisite in that their membrane targeting required both PtdIns(4)P and active Arf1 resulting in a biased localization to the Golgi (Levine and Munro, 2002; Godi et al., 2004; Balla et al., 2005). Similarly, several biosensors developed for PtdIns(3,4,5)P_3_ based on domains of cytohesin-family proteins require Arf/Arl GTPase and/or adjacent polybasic regions for successful membrane targeting (Cohen et al., 2007; Hofmann et al., 2007; Li et al., 2007). Similarly, the PH domain from Bruton's Tyrosine Kinase which recognizes PtdIns(3,4,5)P_3_ can be influenced by direct interaction with heterotrimeric G proteins (Tsukada et al., 1994) and protein kinase C (Yao et al., 1994). The complexities that may arise because of these compounding variables should not fully preclude researchers from utilizing these tools, however. For example, first-generation sensors for PtdIns(4)P provided useful insights to the functions of this lipid in the Golgi, despite being “blind” to other PtdIns(4)P pools (Szentpetery et al., 2010). Likewise, mutations can be introduced into binding domains to prevent protein-protein interactions while preserving PPIns-specificity (Várnai et al., 2005; Cohen et al., 2007; Hofmann et al., 2007; Goulden et al., 2019). Such considerations will be critical in the continued development of improved PPIns biosensors.

### Part III: Tools to Manipulate Phosphoinositides in Leukocytes

Along with the rapid expansion of tools to monitor PPIns came developments that enabled researchers to selectively disrupt these lipids. Many of these experimental approaches can serve “double-duty” by either validating the ability to monitor a specific PPIns pool (Part II) and/or to assess the consequences on downstream effector signaling (Part III). These tools revolve around the manipulation of the kinases, phosphatases, and phospholipases that control phosphoinositide metabolism.

Pharmacological approaches to inhibit PPIns synthesis or degradation are a simple and widely accessible method. As exemplified in Figure 1, membrane-targeted, constitutively-active class I phosphoinositide 3-kinase α (PI3Kα) can be utilized to increase PtdIns(3,4,5)P_3_ in the membrane; this is evinced by the strong membrane enrichment of the AKT PH domain sensor. The addition of the fungal metabolite wortmannin, which can potently inhibit the activity of PI3Ks (Arcaro and Wymann, 1993; Ui et al., 1995), causes the abrupt release of PH-AKT from the membrane (Figure 1B). This simple approach validates the notion that PPIns products of PI3K-activity are required to recruit and retain the biosensor at the plasma membrane, while also demonstrating that the toxin is active against PI3K.

Inhibitors of class I PI3Ks have been a major class of candidates for the treatment of solid and blood-borne cancers. This has resulted in the development of several pan- and isoform-specific class I PI3K inhibitors [reviewed in (Burke, 2018)]. Specific inhibitors have also been developed for several PI4K (Knight et al., 2006; Tóth et al., 2006; Bojjireddy et al., 2014; Li et al., 2017) and PIP5K isoforms (Semenas et al., 2014; Wright et al., 2014). On the other hand, useful inhibitors have also been described for several PPIns phosphatases including those of the SHIP (SH2 domain-containing inositol polyphosphate 5-phosphatase) family which dephosphorylate PtdIns(3,4,5)P_3_ to PtdIns(3,4)P_2_ (Brooks et al., 2010; Fuhler et al., 2012), and several for the INPP5 family (Pirruccello et al., 2014) that dephosphorylate both PtdIns(3,4,5)P_3_ and PtdIns(4,5)P_2_ in the 5-position. The mechanism by which these compounds inhibit SHIP phosphatase activity is unclear, while the INPP5-specific inhibitors bind directly to the catalytic domain. Several PPIns 3- and 4-phosphatases are members of the redox-sensitive protein tyrosine phosphatase family. Oxidizing compounds containing vanadate (e.g., bisperoxovanadate) or the addition of hydrogen peroxide acutely and potently inhibit their activity (Rosivatz et al., 2006; Ross et al., 2007). The acute and reversible nature of this inhibition has been harnessed to understand SAC1 activity in the endoplasmic reticulum (ER) (Zewe et al., 2018) and derivatives have even been applied *in vivo* (Zhang et al., 2017). Lastly, it is possible to deliver PPIns to the cytosol of intact cells by utilizing membrane-permeable acetoxymethyl esterified derivatives. In the cytosol, endogenous esterases cleave the acetoxymethyl group, releasing intact PPIns that then partition into the cytosolic leaflet of organelles.

In addition to pharmacological manipulation, PPIns-metabolizing enzymes can be genetically manipulated by over-expression, RNA interference-mediated depletion, genetic knockout or knock-in mutations, or by exploiting mutations from human samples or model systems. In contrast to pharmacological approaches, these methods are generally chronic in nature and can be susceptible to cellular compensation that may cloud the interpretation. Nonetheless, they represent a valuable way to tease out biological mechanisms when effective and specific pharmacological inhibition is not available for an enzyme of interest (Zunder et al., 2008; Huw et al., 2013). Knock-in mutations incorporated directly into the lipid-binding domains of cellular proteins or PPIns-metabolizing enzymes is a particularly clever way to understand their regulation by phospholipids. Indeed, mutations in amino acids that coordinate the inositol headgroup within biosensors are often included to control for non-lipid-mediated localization (Stauffer et al., 1998; Várnai and Balla, 1998; Várnai et al., 1999, 2005). Conversely, relatively high expression of biosensors or tandem domains of biosensors that have higher avidity can be utilized to effectively occlude downstream signaling by PPIns. Although normally avoided during routine experiments, this approach has been useful in understanding the roles of PtdIns(4,5)P_2_ in controlling cortical actin networks (Raucher et al., 2000; Ueno et al., 2011) and of PtdIns(3)P during resolution of endocytic compartments (Freeman et al., 2019).

PPIns-metabolizing enzymes and their activity can be targeted to virtually any cellular organelle constitutively or acutely to manipulate local PPIns signaling. Constitutive targeting can be accomplished by including defined, well-characterized motifs in the primary sequence of kinases, phosphatases, or phospholipases that dock the enzyme onto the organelle of choice (Figure 1B). Targeting motifs are often transmembrane domains of integral membrane proteins or tail-anchored proteins but electrostatic interactions can also mediate targeting of domains to the plasma membrane (Won et al., 2006; Yeung et al., 2006). PPIns enzyme domains can be recruited to the cytosolic leaflets of specific organelles more acutely (within seconds) by chemically-induced dimerization. The first such system developed was based on the domains from FK506 binding protein (FKBP) and mTOR (FRB domain) that undergo heterodimerization in the presence of rapamycin (Spencer et al., 1993; Inoue et al., 2005). The elegance of this method quickly gained traction for cell biologists as it can allow the tightly controlled depletion of phosphoinositide pools from specific organelles, while largely bypassing any adverse effects of chronic over-expression of PPIns-metabolizing enzymes (Fili et al., 2006; Suh et al., 2006; Varnai et al., 2006; Szentpetery et al., 2010; Hammond et al., 2014). As an alternative to rapamycin-based dimerization, analogous systems have since been developed utilizing the gibberellin plant hormone GA_3_ (Miyamoto et al., 2012), and photoactivation-induced dimerization (Idevall-Hagren et al., 2012). The rate and magnitude of depletion of PPIns can be monitored by co-expression with biosensors and monitoring fluorescence intensity changes or changes in Förster resonance energy transfer (FRET) with fluorophore pairs in the organelle of interest (Varnai et al., 2006; Hammond et al., 2014). In these complex multi-variable experiments, several controls should be implemented for robust conclusions: visualization of the pre- and post-stimulated localization of the protein of interest, recruitment of domains lacking the active cargo (i.e., without the PPIns-metabolizing enzyme) or encoding catalytically-inactive enzymes to control for non-lipid-mediated effects.

Lastly, recent developments have enabled the optogenetic activation of enzymes by incorporating unnatural amino acids (Luo et al., 2014; Courtney and Deiters, 2018). In this case, “caged” (inactive) versions of PPIns-converting enzymes can be expressed in cells at high levels without adverse effects, that can then be activated acutely (Goulden et al., 2019).

## Phosphoinositides in Leukocyte Chemotaxis

The ability of immune cells to migrate is fundamental to embryonic development, infection control, sterile wound healing, the clearance of transformed cells, and tissue regeneration. Its aberrant activation can, however, contribute to inflammatory diseases, tissue necrosis, atherosclerosis, and hematological cancers (Ley et al., 2018; Weavers and Martin, 2020). During chemotaxis, leukocytes extend pseudopods at their leading edge that are directed toward chemoattractants like formyl-peptides, leukotrienes and complement fragments, or away from chemorepellants (Andrew and Insall, 2007; Westman et al., 2019). Through an iterative process, extremely shallow concentration differences (often <5% from leading-to-trailing edge) of the attractants are detected across the plasmalemmal surface and amplified intracellularly. The periodic extension, bifurcation and retraction of leading-edge pseudopodia are driven by dynamic remodeling of the underlying actin cytoskeleton and supported by adherence to the underlying substratum via integrins (Kinashi, 2005; Renkawitz and Sixt, 2010; Weavers and Martin, 2020). In contrast, the trailing edge uropod –which is comprised of more stable actin networks– must simultaneously release from the substratum and retract (Hind et al., 2016). The polarized gliding movement that results can attain speeds >10 μm/min in some leukocytes. The information directing actin polymerization and ongoing feedback for its remodeling are communicated by several parallel pathways involving PPIns that are in turn responsive to the extracellular gradient of the chemoattractants.

Broadly speaking, pseudopod formation requires activation of the Rho-family GTPases Rac and Cdc42 to drive F-actin polymerization into protrusions that drive forward motion (Kraynov et al., 2000; Itoh et al., 2002; Ridley et al., 2003; Willard and Devreotes, 2006; Yang et al., 2016). Conversely, the sides and uropod contain active RhoA, myosin light chain kinase, and ezrin-radixin-moesin (ERM) protein scaffolding to support actomyosin-based contraction of the trailing edge and stabilize adhesion to the endothelium during extravasation (Yoshinaga-Ohara et al., 2002; Xu et al., 2003; Lee et al., 2004a; Hind et al., 2016).

### Pseudopod Organization by 3-Phosphorylated Inositides

**Figure 2:**
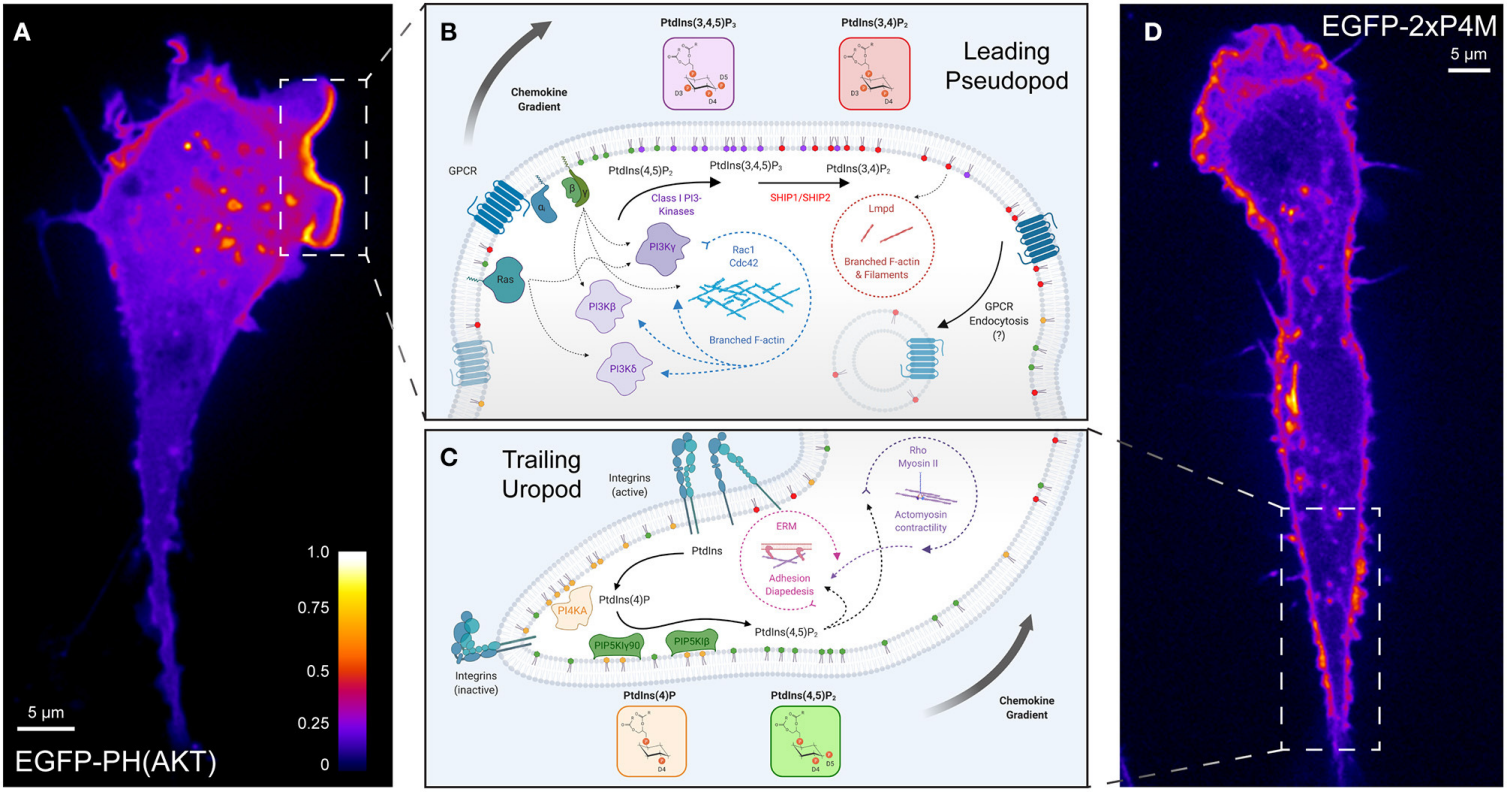
Polarization of phosphoinositide signals during chemotaxis. **(A)** A representative confocal micrograph of PH-AKT expressed in a RAW264.7 monocytic cell undergoing chemokinesis (random motion) in the presence of growth factors from fetal-bovine serum. PH-AKT bi-specifically recognizes PtdIns(3,4,5)P_3_ and PtdIns(3,4)P_2_. The image has been pseudocolored to reflect the abundance of the probe. **(B)** Phosphoinositide signaling controlling “frontness” during leukocyte chemotaxis. A signal arising from the stimulation of cell surface GPCRs and associated activation of G-proteins (β, γ) by chemokines lead to the activation of class I PI3Ks to produce PtdIns(3,4,5)P_3_ from PtdIns(4,5)P_2_. Together with PtdIns(3,4)P_2_, the product of dephosphorylation of PtdIns(3,4,5)P_3_, 3-phosphoinositides in the leading-edge membrane mediate a feed-forward loop that activates Rho- and Arf-family GTPases and Lmpd to drive actin polymerization and locally amplify PI3K-signaling. **(C)** Engagement of substratum by leukocytes favors the “backness” signals PtdIns(4)P and PtdIns(4,5)P_2_. PI4KA mediates the synthesis of PtdIns(4)P from PtdIns, which is necessary for the activation of PIP5KIγ90 and the generation of PtdIns(4,5)P_2_. RhoA signaling networks and ERM proteins regulated by PtdIns(4,5)P_2_ are critical for leukocyte rolling adhesion, diapedesis, and uropod contraction during migration. **(D)** A representative confocal micrograph of 2xP4M expressed in a RAW264.7 cell undergoing chemokinesis (random motion) in the presence of growth factors from fetal-bovine serum. The P4M domain derived from *Legionella*'s SidM specifically recognizes PtdIns(4)P. The image has been pseudocolored to reflect the abundance of the probe in the Golgi, endolysosomes, and the plasma membrane including in the region of the uropod. GPCR, G protein-coupled receptor; PI3K, phosphoinositide 3-kinase; SHIP, SH2 domain-containing inositol polyphosphate 5-phosphatase; Lmpd, Lamellipodin; ERM, Ezrin-Radixin-Moesin.

The 3-phosphorylated species PtdIns(3,4,5)P_3_ and PtdIns(3,4)P_2_ are markedly enriched at the leading edge of migrating cells (Figure 2A). This hallmark of polarized migration has been recognized across numerous subsets of leukocytes, though the first identification occurred in the social amoebae *Dictyostelium* (Meili et al., 1999; Dormann et al., 2002). Robust signaling through class I PI3Ks is the principal determinant of accumulation of 3-phosphorylated species at the leading edge. Of note, PtdIns(3,4,5)P_3_ and/or PtdIns(3,4)P_2_ are polarized toward chemoattractants despite depolymerization of the underlying actin cytoskeleton (Servant et al., 2000; Dormann et al., 2002; Janetopoulos et al., 2004; Xu et al., 2005) demonstrating that gradient sensing and PPIns polarization are upstream of the cytoskeletal rearrangement and morphological changes. Evidence for the role of PI3Ks in chemotaxis came from pharmacological treatment with wortmannin or LY294002, which effectively block PtdIns(3,4,5)P_3_ production and recruitment of PH-AKT to the inner leaflet of the PM in response to several chemoattractants (Knall et al., 1997; Niggli and Keller, 1997; Servant et al., 2000). Further, the importance of 3-phosphorylated species has been highlighted by the sufficiency of exogenously delivered PtdIns(3,4,5)P_3_ (Niggli, 2000; Weiner et al., 2002) or the synthetic activation of endogenous PI3Ks (Inoue and Meyer, 2008) to polarize neutrophils, signal downstream actin polymerization, and elicit random leukocyte migration.

Class I PI3Ks are activated in response to chemokines via two major pathways: signaling through G protein βγ subunits liberated from αi downstream of activated GPCRs, and by the small GTPase Ras through Ras-binding domains in several PI3Ks (Figure 2B, left) (Suire et al., 2006; Kurig et al., 2009; Surve et al., 2014). Of the four class I PI3K isoforms which are expressed in leukocytes, the class IB isoform PI3Kγ was identified as the chief kinase generating PtdIns(3,4,5)P_3_ in response to chemotactic stimuli in leukocytes. Neutrophils and macrophages, natural killer (NK) lymphocytes, and T lymphocytes that are deficient in PI3Kγ do not migrate efficiently toward various chemoattractants *in vitro* or to sites of inflammation *in vivo* (Hirsch et al., 2000; Li et al., 2000; Sasaki et al., 2000; Hannigan et al., 2002; Reif et al., 2004; Suire et al., 2006; Ferguson et al., 2007; Nishio et al., 2007; Saudemont et al., 2009). Interestingly, substantial positive crosstalk exists between PI3K, its initial PtdIns(3,4,5)P_3_ synthesis, and cytoskeletal regulators. A secondary activation of PI3Ks has been posited to amplify and sustain this important signaling node during chemotaxis (Niggli, 2000; Sadhu et al., 2003; Boulven et al., 2006). One way this occurs is by initiating a feedback loop between PI3Ks and Rho-family GTPases (Figure 2B, center circle), which activates additional PtdIns(3,4,5)P_3_ synthesis (Servant et al., 2000; Wang et al., 2002; Weiner et al., 2002; Srinivasan et al., 2003; Park et al., 2004; Inoue and Meyer, 2008; Kuiper et al., 2011). The pre-treatment of cells with *Clostridioides-*derived toxins (which inactivate several Rho-family GTPases) or interference with Rho-family activating proteins can strongly reduce the polarization of PH-AKT in response to chemokines (Weiner et al., 2002; Srinivasan et al., 2003; Kunisaki et al., 2006). How does this occur mechanistically? Both active Rac1 and Cdc42 can directly associate with PI3Kβ and stimulate its lipid kinase activity (Fritsch et al., 2013). Additionally, the activation of PI3Kβ by G-protein βγ subunits (Figure 2B, center) when GPCRs and receptor tyrosine kinases (RTKs) are co-stimulated (Houslay et al., 2016) could mechanistically explain the contribution of PI3Kβ to leukocyte migration (Vanhaesebroeck et al., 1999; Ferguson et al., 2007). In parallel, genetic or pharmacological inhibition of the hemopoietic-specific class IA PI3Kδ revealed a pronounced role of this isoform in PtdIns(3,4,5)P_3_ synthesis, polarization, and directed migration of neutrophils, lymphocytes, and NK cells (Sadhu et al., 2003; Reif et al., 2004; Saudemont et al., 2009). The activation of PI3Kδ is likely secondary to the PI3Kγ-mediated activation of Rho-family effectors or occurs downstream of Ras activation (Figure 2B) (Burke, 2018). Importantly, despite similar enzymatic activity, distinct PI3K isoform-specific roles have been revealed *in vivo*: PI3Kγ mediates early extravasation and chemotaxis, while PI3Kδ sustains long-term chemotaxis into inflamed tissues (Liu et al., 2007). Lastly, effectors of Rac1 recruited by the products of PI3K (discussed further below) can in turn support the polarization of 3-phosphorylated inositides in the pseudopod (Kunisaki et al., 2006).

Beyond the edges of the leading pseudopod, PtdIns(3,4,5)P_3_ is limited in abundance and distribution by the PPIns 3-phosphatase, PTEN (phosphatase and tensin homolog) (Ferguson et al., 2007; Nishio et al., 2007), and by the action of the Type III 5-phosphatases SHIP1 (Nishio et al., 2007) and SHIP2 (Lam et al., 2012). Collectively, the 5-phosphatases convert PtdIns(3,4,5)P_3_ to PtdIns(3,4)P_2_, while PTEN terminates signaling by hydrolyzing both PtdIns(3,4,5)P_3_ and PtdIns(3,4)P_2_ to generate PtdIns(4,5)P_2_ and PtdIns(4)P, respectively (Malek et al., 2017; Goulden et al., 2019). Although data on the role of PTEN in mammalian leukocytes is somewhat discrepant (Nishio et al., 2007; Wang, 2009; Balla, 2013), the phosphatase has been localized to the trailing uropod of neutrophils (Li et al., 2005), similar to its polarized localization in *Dictyostelium* (Iijima and Devreotes, 2002). The depletion of PTEN in both systems causes abnormal actin polymerization into multiple pseudopods and prolongs the duration of AKT signaling (Funamoto et al., 2002; Iijima and Devreotes, 2002; Huang et al., 2003; Subramanian et al., 2007; Li et al., 2019). Although the resulting migration is error-prone and often fails to prioritize between chemotactic signals, migration speed actually increases, augmenting the number of PTEN-null neutrophils that enter inflamed tissues *in vivo* (Subramanian et al., 2007; Heit et al., 2008b; Sarraj et al., 2009). Recruitment of PTEN to the membrane, which is critical for its lipid phosphatase activity, occurs largely through its interaction with PtdIns(4,5)P_2_ (Rahdar et al., 2009), but also by front-to-back signaling networks involving PI3Kδ and RhoA (Li et al., 2005; Papakonstanti et al., 2007) (Figure 2C). Unlike *Dictyostelium*, mammalian cells are also regulated by SHIP1 and SHIP2. The deletion of SHIP1 in neutrophils and macrophages severely inhibits their speed and ability to polarize their actin cytoskeleton toward various chemoattractants *in vitro*, phenocopying cells lacking PI3Kγ (Ferguson et al., 2007; Nishio et al., 2007). PtdIns(3,4,5)P_3_ levels are elevated at rest and during stimulation in these cells and SHIP1^−/−^ cells have multiple broad, distorted lamellae marked by the AKT biosensor. This implies an important regulatory role for SHIP phosphatases and their enzymatic activity in organizing the pseudopod (Nishio et al., 2007).

It is nevertheless important to note that although PI3Ks and their downstream products are critical for many aspects of chemotaxis –such as speed and initiating morphological polarization—PI3Ks do not comprise the basis for the “biological compass” that orients cells toward or away from the chemical stimulus itself. In many settings, the deletion or inhibition of PI3Ks does not ultimately eliminate the ability of cells to bias their motility in the direction of a chemotactic signal; PI3Ks merely help to get them there (Loovers et al., 2006; Hoeller and Kay, 2007; Nishio et al., 2007; Takeda et al., 2007; Heit et al., 2008a). Studies of leukocyte recruitment *in vivo* have revealed that several other pathways operate in parallel or in conjunction with PI3K-related pathways to properly resolve the complex collective of endogenous and exogenous chemotactic signals (Heit et al., 2002, 2008a,b).

### PtdIns(4)P, PtdIns(4,5)P_2_, and the Control of “Backness”

In contrast to 3-phosphorylated species, PtdIns(4)P and PtdIns(4,5)P_2_ are sustained in an opposing back-to-front gradient (Figures 2C,D) which has important consequences for extravasation and to establish the “biological compass” of migrating leukocytes. In addition to its phosphorylation by class I PI3Ks, PtdIns(4,5)P_2_ is selectively hydrolyzed at the leading edge by PLC. The activation of G proteins βγ by chemokines triggers several isoforms, including PLCβ2 and PLCβ3, to be activated at the leading edge of migrating leukocytes (Tang et al., 2011; Balla, 2013). PLCβs possess N-terminal PH domains that interact with PtdIns(4,5)P_2_ and Ins(1,4,5)P_3_, as well as a polybasic C-terminal region (Balla, 2013) that likely favors association with negatively charged lipids [i.e., PtdIns(3,4,5)P_3_] at the leading edge. Interestingly, PLCβ2 is also regulated by Rho-family GTPases, as exemplified by its binding to Rac and sequestration into subdomains of the PM (Illenberger et al., 2003; Gutman et al., 2010; Tang et al., 2011). Together, these membrane-targeting mechanisms support PLC-mediated hydrolysis of PtdIns(4,5)P_2_ to diacylglycerol (DAG) and Ins(1,4,5)P_3_ in the pseudopod (Keizer-Gunnink et al., 2007; Nishioka et al., 2008)—two intermediates with important consequences on the activation of integrin-based adhesiveness (Kinashi, 2005; Herter and Zarbock, 2013). In combination with PI3K activity, PLCβ2 and β3 enzymes are clearly important for establishing the back-to-front gradient of RhoA signaling and myosin contractility in leukocytes (Gao et al., 2015), which ultimately impact chemotaxis greatly (Tang et al., 2011).

Within the uropod, several type I PIP5Ks (PIP5KI) are activated to generate a modest enrichment of PtdIns(4,5)P_2_, which can be visualized with the biosensor PH-PLCδ (Figure 2C) (Lokuta et al., 2007; Xu et al., 2010). The engagement of α_L_β_2_ and α_M_β_2_-integrins triggers the polarization of PIP5KIγ90 (also called PIP5K1C90) to the uropod of migrating cells (Xu et al., 2010), likely supported by the ability of the kinase to bind anionic lipids within the PM (Fairn et al., 2009). PIP5KIβ also has been localized to the uropod of migrating leukocytes, supported by its interaction with ERM proteins (Lacalle et al., 2007; Mañes et al., 2010). PtdIns(4,5)P_2_ produced by PIP5KIs was initially posited to be sufficient to control “backness” by positively-regulating RhoA-signaling and ERM-mediated linkage to the plasma membrane (Xu et al., 2010) –both critical features of the uropod (Figure 2C, center). However, more recently it was realized that the PIP5KI-mediated synthesis of PtdIns(4,5)P_2_ is accompanied by an enrichment of its substrate, PtdIns(4)P, within the uropod (Ren et al., 2019) (Figure 2D). An innovative study by Ren *et al*. revealed that not only is plasmalemmal PtdIns(4)P polarized toward the uropod during extravasation but depleting the inositide destroys the polarization to the uropod of several proteins including PIP5KIγ90 itself and active myosin light chain. The resulting PtdIns(4)P-depleted neutrophils are defective in their ability to bind to inflamed endothelium as a result of these polarization defects.

PtdIns(4)P is maintained in multiple sub-cellular compartments including the Golgi, late endosomes/lysosomes, and the PM by the activity of four PI4-kinases (Balla, 2013; Hammond et al., 2014). In the uropod membrane, PtdIns(4)P is synthesized by PI4KA following its activation by srGAP, an inverted F-BAR protein that senses increased membrane curvature (Ren et al., 2019). These studies present an interesting paradigm in which the polyanionic lipids PtdIns(4)P and PtdIns(4,5)P_2_ positively influence each other, orchestrate the stereospecific and electrostatic recruitment of effector proteins that scaffold the uropod, and ultimately the adhesion and initial directionality of leukocyte migration.

### Phosphoinositide Effectors During Chemotaxis

The actin cytoskeleton receives multiple inputs via PPIns. One important cytoskeletal effector at the leading edge is the five-membered WAVE (SCAR/WASP family verprolin-homologous protein) regulatory complex. WAVE is one of several nucleation-promoting factors (NPFs) necessary for the full activation of the Arp2/3 complex that generates branching actin filaments (Takenawa and Suetsugu, 2007). The importance of WAVE for migration is supported by numerous studies in different cell types and organisms (Krause and Gautreau, 2014) including leukocytes, where WAVE complex members rapidly localize to the leading pseudopod (Weiner et al., 2006, 2007; Millius et al., 2009). Although the WAVE complex can be recruited and activated directly by receptors (including possibly the CXCR5 chemokine receptor) (Chen et al., 2014), this complex is generally recruited and activated at the membrane by factors such as lipids. Within the WAVE complex, WAVE1, WAVE2, and WAVE3 possess a carboxy-terminal basic region that has a higher affinity for PtdIns(3,4,5)P_3_ over other inositides and can promote its membrane recruitment (Oikawa et al., 2004). Normally inhibited *in trans* by other complex members (Eden et al., 2002), the WAVE complex can be activated by GTP-bound Rac on PtdIns(3,4,5)P_3_-containing liposomes (Lebensohn and Kirschner, 2009) and by Arf GTPases that synergize with Rac in the presence of this inositide (Koronakis et al., 2011).

Supporting these notions, a number of guanine nucleotide exchange factors (GEFs) and GTPase-activating proteins (GAPs) for Rho-, Arf-, and Ras-families of GTPases are recruited to membrane domains by PPIns-binding domains that recognize PtdIns(3,4,5)P_3_ and/or PtdIns(3,4)P_2_ (Krugmann et al., 2002; Rossman et al., 2005; Campa et al., 2015; McCormick et al., 2019). GEFs aid in the exchange of GDP for GTP, thereby promoting effector association, while GAPs enhance their intrinsically low GTPase activity. Therefore, the recruitment of Rac, Cdc42, and Arf GEFs and GAPs to the leading edge can indirectly regulate effectors of cytoskeletal remodeling. Prototypical examples have been reported for several Rac GEFs such as Vav1/3, Tiam1/2, and P-rex1, which are recruited to the pseudopod membrane in a PI3K-dependent manner to stimulate chemotaxis via Rac [see (McCormick et al., 2019) and (Campa et al., 2015)]. Leukocytes also express several atypical Rac GEFs from the Dock family that function through association with Elmo proteins (Sanui et al., 2003). These bipartite GEFs specifically associate with PtdIns(3,4,5)P_3_ for activation (Côté et al., 2005) but, conversely, are also required for full PI3K activation and 3-PPIns polarization during chemotaxis (Kunisaki et al., 2006). Similarly, the recruitment to the pseudopod of several Arf GEFs, including ARAP3 (Krugmann et al., 2002; Gambardella et al., 2013) and GBF1 (Mazaki et al., 2012), occurs via 3-PPIns.

The recruitment and activation of the WAVE complex is independently promoted by PtdIns(3,4)P_2_ and its binding partner, lamellipodin (Lmpd) (Figure 2B, right). Initially described during fibroblast migration, Lmpd recruitment to activated RTKs is dictated by its PH domain, that has affinity for PtdIns(3,4)P_2_, and by its Ras-association domain which can interact with both active Ras and Rac (Krause et al., 2004; Law et al., 2013); these determinants promote the direct interaction between Lmpd and the WAVE complex at the leading edge that controls migration speed and directional persistence. Lmpd can also promote actin filament elongation at the leading edge by recruiting Ena/VASP proteins (Krause et al., 2004; Michael et al., 2010; Hansen and Mullins, 2015; Carmona et al., 2016). This molecular axis has since been extended to other settings which include leukocyte migration: the depletion of PtdIns(3,4)P_2_ by overexpressing the PPIns 4-phosphatases INPP4A/B severely inhibits the migration speed and ability of lymphocytes to orient toward chemokines (Li et al., 2016). The details of how Lmpd is activated downstream of G proteins in leukocytes is unclear, but a mechanism can be gleaned by analogy with its activation by RTKs. Not only are class IA PI3Ks and SHIP2 activated by RTKs to produce PtdIns(3,4,5)P_3_ and PtdIns(3,4)P_2_, respectively, but so too are several GEFs for Rac and Ras GTPases. Lmpd could sense similar inputs downstream of GPCR activation.

Lastly, in addition to directly regulating the actin cytoskeleton, a tantalizing possible function of PtdIns(3,4)P_2_ and Lmpd in leukocyte migration may be that they regulate the selective endocytosis of activated GPCRs via a pathway termed Fast Endophilin-Mediated Endocytosis, or FEME for short (Boucrot et al., 2015) (Figure 2B, right). This clathrin-independent pathway relies on the localized synthesis of PtdIns(3,4)P_2_ sensed by Lmpd, to engage endophilin and pre-localize this endocytic complex at the leading edge of migrating cells (Chan Wah Hak et al., 2018). In the event of receptor activation, PtdIns(3,4)P_2_ synthesis can trigger the downregulation of PI3K-signaling by endocytosis of activated cell surface receptors. Although the FEME pathway is active in lymphocytes, the role of endophilin and FEME in leukocyte chemotaxis and GPCR trafficking are yet to be explored.

### Pathogens Interfere With Phosphoinositide Signaling During Chemotaxis

Considering the fundamental role that PPIns play in various cellular processes, it is not surprising that certain pathogens have developed strategies to hijack inositide signaling to create or sustain their replicative niche (Kumar and Valdivia, 2009; Pizarro-Cerdá et al., 2015; Walpole and Grinstein, 2020). The deployment of PPIns-specific metabolizing kinases and phosphatases into the host cell by several pathogens is one exemplary case.

The Gram-negative obligate anaerobe *Treponema denticola* (*T. denticola*) is a key bacterial pathogen in the development of oral periodontitis (Sela, 2001), the leading cause of tooth-loss worldwide (Darveau, 2010). In addition, periodontitis has been increasingly implicated as a driver of other systemic diseases, underscoring the importance of understanding its pathogenesis. *T. denticola* is normally a minor component of the diverse microbial community within the oral cavity, but can opportunistically take hold during dysbiosis and contribute to the inflammation-mediated breakdown of soft tissues, bone resorption, and resulting tooth loss. Following its attachment to the extracellular matrix, the spirochete expresses a major outer membrane sheath protein known as Msp that targets PI3K-signaling in neutrophils. Specifically, Msp reduces neutrophil PI3K activity (Visser et al., 2013) and hyperactivates PTEN (Jones et al., 2019), reducing cellular PtdIns(3,4,5)P_3_ and PtdIns(3,4)P_2_ levels. Consistent with its hyperactivation, PTEN is constitutively recruited to the plasma membrane in Msp treated-neutrophils (Jones et al., 2019). Because of the aberrant PPIns signaling, Msp potently blocks the activation of Rac1 and precludes the necessary actin rearrangements that drive effective chemotaxis (Thomas et al., 2006; Jones et al., 2019). The C-terminus of Msp is necessary for such effects (Jones et al., 2017), but how or if the effector is delivered into the host cell cytosol to manipulate PPIns-metabolizing enzymes remains an unresolved question in periodontal research.

## Phosphoinositides During Phagocytosis

Phagocytosis is the process whereby cells internalize and dispose of solid particles. Specific cell surface receptors recognize phagocytic targets and deliver them into vacuoles known as phagosomes. Phagocytosis plays essential roles throughout the body and can be carried out by multiple cell types. Phagocytosis carried out by myeloid cells such as macrophages, neutrophils and dendritic cells, constitutes the first line of defense against invading microorganisms and is also essential for the development of the adaptive immune response through antigen presentation. These myeloid cells are collectively known as professional phagocytes. Secondly, phagocytosis is fundamental for the daily clearance of billions of apoptotic cells, maintaining homeostasis within an organism. Professional, as well as non-professional phagocytes such as fibroblasts, epithelial, endothelial and mesenchymal cells, can clear apoptotic cells. Finally, phagocytosis of effete cells plays a pivotal role in wound healing, tissue development, morphogenesis and regeneration. The elimination of effete cells is carried out by both professional and non-professional phagocytes.

Given this variety of biological functions and the myriad phagocytic ligands, a sizeable number of receptors are required to recognize and discriminate the diversity of phagocytic targets (Flannagan et al., 2012). Amongst these receptors are: (1) pattern-recognition receptors (PRRs) like MARCO that bind pathogen-associated molecular patterns (PAMPs) present on microbial surfaces; (2) receptors like TIM-4 that bind phosphatidylserine and other apoptotic corpse markers; and (3) opsonic receptors such as FcɤR and iC3b that recognize immunoglobulin-opsonized pathogens or complement-opsonized foreign and self-antigens, respectively. The most studied of these is by far the Fcɤ receptor family, which we will use as a prototype throughout this review.

The diversity of phagocytic targets and receptors entails patently different molecular mechanisms of phagosome formation, maturation and resolution. Despite these differences, all types of phagocytosis share an inherent dependence on the rearrangement of the actin cytoskeleton and on the dynamic remodeling of the plasma membrane, as the phagosome evolves.

Phagocytosis can be divided into three main stages: phagosome formation, phagosome maturation and phagosome resolution. The formation of the phagosome involves probing for potential targets by plasma membrane ruffling, followed by target binding, pseudopod progression around the target, and scission of the phagosome from the plasma membrane (Hoppe and Swanson, 2004; Levin et al., 2016). During the maturation stage nascent phagosomes convert into early phagosomes that in turn evolve into late phagosomes and then to phagolysosomes (Vieira et al., 2002; Canton, 2017; Levin et al., 2017). The ultimate resolution of phagolysosomes entails their shrinkage and recycling of membrane and luminal components (Levin-Konigsberg et al., 2019).

**Figure 3:**
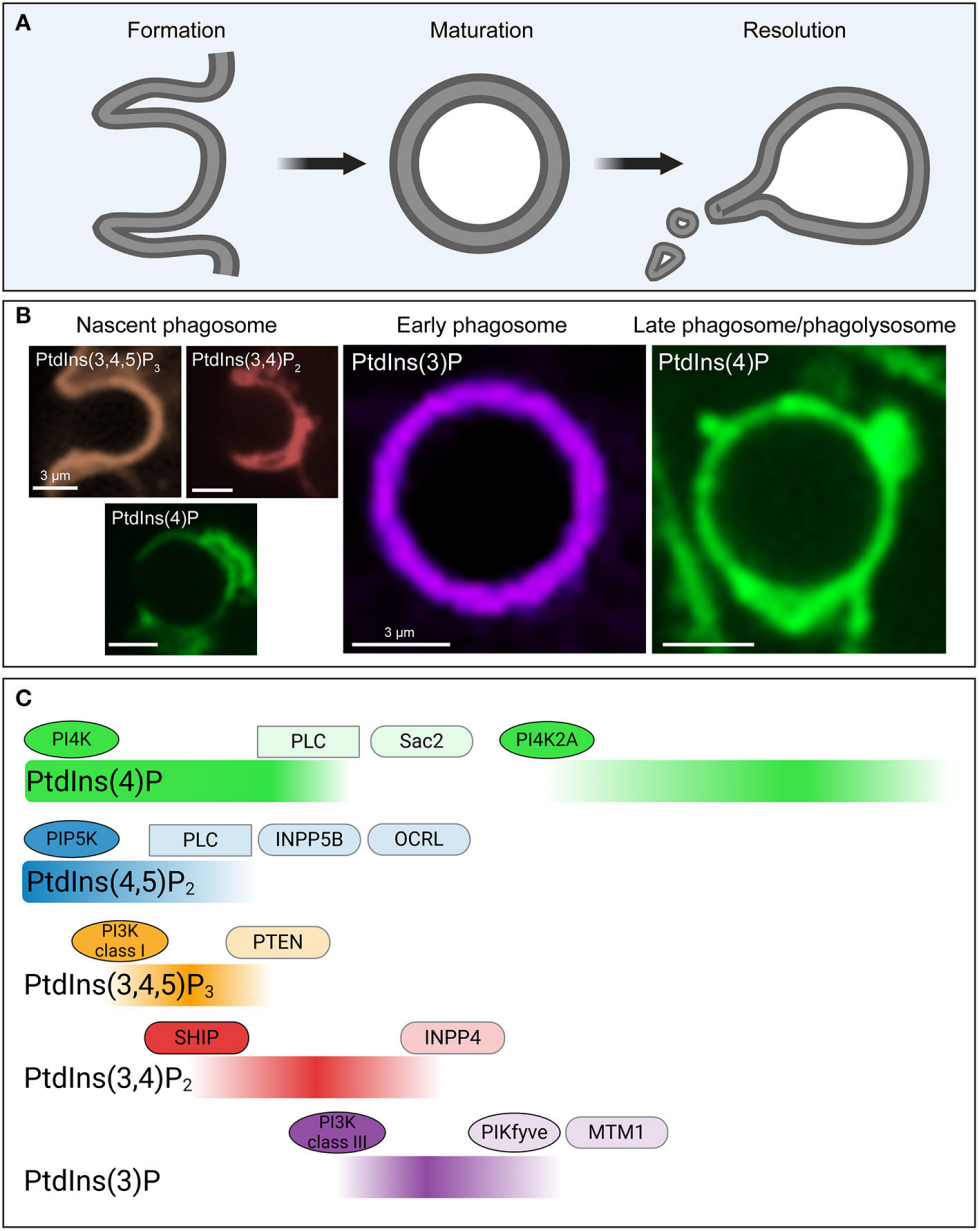
Phosphoinositide fluxes drive phagosome formation, maturation, and resolution. **(A)** Graphic representation of the process of phagocytosis. The three main stages (phagosome formation, phagosome maturation and phagosome resolution) are depicted. **(B)** Representative confocal micrographs of some of the biosensors used to detect phosphoinositides during the three main stages of phagocytosis, color-coded to match **(C)**. **(C)** Temporal distribution of five major phosphoinositides and the enzymes involved in their metabolism during phagosome formation, maturation and resolution. During these stages the levels of PtdIns4P (green), PtdIns(4,5)P_2_ (blue), PtdIns (3,4,5)P_3_ (orange), PtdIns(3,4)P_2_ (red), and PtdIns(3)P (purple) in the cytosolic leaflet of the phagosome undergo drastic changes, as indicated. These changes are mediated by a series of kinases (ovals), phosphatases (rounded rectangles/capsules), and phospholipases (rectangles) that accumulate and are activated at the phagosomal membrane at distinct timepoints during the process of phagocytosis. PI4K, phosphoinositide 4-kinase; PI3K, phosphoinositide 3-kinase; PLC, phospholipase C; SHIP, SH2 domain containing inositol polyphosphate 5-phosphatase; INPP, inositol polyphosphate phosphatase; OCRL, oculocerebrorenal Lowe syndrome protein; PTEN, phosphatase and tensin homolog; MTM, myotubularin.

Phosphoinositides in the cytosolic leaflet of the phagosomal and plasma membrane orchestrate the changes in membrane composition and actin cytoskeleton during each stage of phagocytosis. The phosphoinositides with documented essential roles during phagocytosis are PtdIns(3)P, PtdIns(4)P, PtdIns(4,5)P_2_ and PtdIns(3,4,5)P_3_ (Bohdanowicz and Grinstein, 2013; Swanson, 2014; Levin-Konigsberg et al., 2019); these will therefore occupy center-stage in this section of the review. The following pages describe the dynamics of phosphoinositides during phagosome formation, maturation and resolution (Figure 3).

## Phagosome Formation

The formation of the phagosome can be divided into three main stages: (1) Ruffling and probing for targets; (2) binding of the target particle and pseudopod progression, and (3) phagosomal scission. The following pages provide a detailed description of the role of phosphoinositides during these sub-stages.

1. Ruffling and target probing: elevated PtdIns(4,5)P_2_ and PtdIns(3,4,5)P_3_.

Phagocytic cells, such as macrophages and dendritic cells, constantly probe for targets by ruffling their plasma membrane and extending pseudopods (Bohdanowicz et al., 2013). As described in the chemotaxis section, these membranous protrusions are driven by actin polymerization, which is facilitated by elevated PtdIns(4,5)P_2_ levels. Additionally, accumulation of PtdIns(3,4,5)P_3_ and PtdIns(3,4)P_2_ at the leading edge of ruffling membranes control actin assembly and disassembly. PtdIns(4)P is also present in the plasma membrane of resting phagocytes and during phagosome formation, however its role during this stage is undefined.

At rest, PtdIns(4,5)P_2_ is localized in the cytosolic leaflet of the plasma membrane primarily, where it accounts for about 1–2% of the total phospholipid content (McLaughlin and Murray, 2005). The elevated levels of PtdIns(4,5)P_2_ in ruffling membranes are generated by PIP5KI, which phosphorylate PtdIns(4)P at the D5 position of the inositol ring. Type II phosphatidylinositol phosphate kinases could conceivably phosphorylate PtdIns(5)P at the D4 position and generate PtdIns(4,5)P_2_. Moreover, dephosphorylation of PtdIns(3,4,5)P_3_ by PTEN would also yield PtdIns(4,5)P_2_. However, the contribution of the these latter pathways to PtdIns(4,5)P_2_ formation in the resting state is thought to be insignificant (Mondal et al., 2011; Bohdanowicz and Grinstein, 2013) (Figure 3).

The increased activity of PIP5KI observed during ruffling is triggered, at least partially, through stimulation by Rho-family (Tolias et al., 1995; Weernink et al., 2004) and Arf GTPases (Honda et al., 1999; Brown et al., 2001). PtdIns(4,5)P_2_, can in turn stimulate Rho GTPases, making this positive regulation reciprocal (Tolias et al., 2000). Elevated levels of phosphatidic acid have been reported in the plasma membrane of ruffling phagocytic cells (Bohdanowicz et al., 2013), where it is thought to activate PIP5KI via Arf6 (Honda et al., 1999). Remarkably, production of phosphatidic acid from phosphatidylcholine by PLD is also dependant on PtdIns(4,5)P_2_ as a cofactor (Divecha et al., 2000). Accordingly, a constitutive positive feedback loop between Rho and Arf GTPases, phosphatidic acid, and PIP5KI allows for continuous probing by resting phagocytic cells.

Actin polymerization in ruffles and pseudopods is coordinated by elevated levels of PtdIns(4,5)P_2_ in multiple ways. Firstly, PtdIns(4,5)P_2_ provides stability to the active state of NPFs such as WASP and N-WASP, members of the of the Wiskott-Aldrich syndrome protein (WASP) family (Rohatgi et al., 2000, 2001). NPFs activate the Arp2/3 complex, which in turn catalyzes branched actin filament nucleation (May et al., 2000). PtdIns(4,5)P_2_-mediated WASP stabilization was also shown to be dependent on Rho GTPases (Caron and Hall, 1998; May et al., 2000; Park and Cox, 2009). Furthermore, PtdIns(4,5)P_2_ can directly activate formins, a family of linear actin nucleators (Rousso et al., 2013). Secondly, PtdIns(4,5)P_2_ inhibits actin-severing proteins, such as gelsolin (Janmey and Stossel, 1987) and cofilin (Gorbatyuk et al., 2006), thus curtailing the depolymerization of the actin cytoskeleton. Thirdly, PtdIns(4,5)P_2_ allows for the growth of actin filaments by recruiting I-BAR proteins to the tip of the pseudopods, fostering actin polymerization (Hotulainen and Saarikangas, 2016). Lastly, PtdIns(4,5)P_2_ facilitates the tethering of the plasma membrane to the underlying actin cytoskeleton through the ERM family of anchor proteins (Bretscher et al., 2002).

Elevated basal levels of PtdIns(3,4,5)P_3_ have also been reported in the plasma membrane of probing phagocytic cells (Bohdanowicz et al., 2013; Canton et al., 2016), allowing for the rearrangement of the actin cytoskeleton necessary for pseudopod protrusion and increased phagocytic receptor mobility. As described earlier in this review, PtdIns(3,4,5)P_3_ production primarily occurs through the phosphorylation of PtdIns(4,5)P_2_ by class I PI3Ks. Mechanisms by which PtdIns(3,4,5)P_3_ can promote actin rearrangement are described in the following section but share similarities with proposed functions in chemotaxis.

2. Target binding: high PtdIns(4,5)P_2_ and appearance of PtdIns(3,4,5)P_3_ in the phagocytic cup and Pseudopod progression: decline of PtdIns(4,5)P_2_ with sustained PtdIns(3,4,5)P_3_.

Ruffling and probing increase the probability of contacting a phagocytic target. Phagocytosis is initiated upon the binding of phagocytic target ligands to one or more receptors expressed on the surface of phagocytic cells. During the first stage of phagocytosis PtdIns(4,5)P_2_ transiently rises, whereas a marked accumulation of PtdIns(3,4,5)P_3_ occurs at the newly formed phagocytic cup.

Ligation of multiple vicinal ligands causes receptor clustering and activation (Jones et al., 1985). In the case of Fcɤ receptors, clustering prompts the phosphorylation of their immunoreceptor tyrosine-based activation motifs (ITAMs) by non-receptor tyrosine kinases of the Src family (Ghazizadeh et al., 1994), which include Lyn and Hck (Wang et al., 1994; Carréno et al., 2002). Phosphorylated ITAMs interact with Syk kinase which has two Src-homology two domains (Greenberg et al., 1996). Syk then recruits and phosphorylates scaffolding adaptors like Gab2, which in turn recruit p85, the catalytic domain of PI3K to the site of receptor binding (Gu et al., 2003). PI3K catalyzes the formation of PtdIns(3,4,5)P_3_ from PtdIns(4,5)P_2_, and important signal for the progression and completion of phagocytic cup formation (Figure 3).

Concomitant with the synthesis of PtdIns(3,4,5)P_3_ is the formation of its precursor PtdIns(4,5)P_2_. Extending pseudopods exhibit a moderate elevation of PtdIns(4,5)P_2_ relative to resting plasma membrane. Increased production rather than reduced consumption explains this elevation. PIP5KI activity is stimulated by elevated phosphatidic acid levels catalyzed by the enhanced activity of PLD (Divecha et al., 2000) observed during phagocytosis (Kusner et al., 1999; Lee et al., 2002; Iyer et al., 2004). In addition, activated Rho GTPases downstream of engaged phagocytic receptor signaling sustain the activity of PIP5KI (Fairn et al., 2009). Together, these effectors elevate PtdIns(4,5)P_2_ in the periphery of forming phagocytic cups (Botelho et al., 2000; Hoppe and Swanson, 2004).

Upon particle engagement and receptor clustering, the extending pseudopods wrap around the phagocytic target in a zipper-like manner. Two opposing modalities of actin dynamics favor the expansion of the contact area with the phagocytic target. Initially, branched and linear actin filament polymerization is required to drive the plasma membrane around the particle. Conversely, actin disassembly must occur at the base of the phagocytic cup; otherwise, polymerised actin would act as a mechanical obstacle to proper engulfment (O'Callaghan et al., 2011), preventing delivery of endomembranes (Bajno et al., 2000; Dewitt et al., 2006) and restricting receptor clustering at the phagocytic synapse (Treanor et al., 2011; Freeman et al., 2015). While PtdIns(4,5)P_2_ is known to facilitate the growth of actin filaments in a manner analogous to the one described above for the ruffling membranes, PtdIns(3,4)P_2_ can also coordinate actin assembly. PtdIns(3,4)P_2_ formed by the dephosphorylation of PtdIns(3,4,5)P_3_ is known to recruit Lamellipodin and thereby ENA/VASP to the leading edge of migrating cells (Krause et al., 2004) and could tentatively play an analogous role during phagosome formation. In line with this, Ena/VASP family proteins seem to be essential for the FcγR receptor-mediated remodeling of the actin cytoskeleton (Coppolino, 2001) (Figure 3).

PtdIns(4,5)P_2_ then disappears from the base of the phagocytic cup, coinciding in time and space with the disassembly of cortical actin (Scott et al., 2005). PtdIns(4,5)P_2_ disappearance from the base of the phagocytic cup can be attributed to three main factors. Firstly, PtdIns(4,5)P_2_ is consumed by PI3K when producing PtdIns(3,4,5)P_3_. Secondly, PtdIns(3,4,5)P_3_ recruits PLC (see Table 1) to the phagosome where it hydrolyzes PtdIns(4,5)P_2_ (Falasca et al., 1998) and is likely to represent the main factor responsible for the disappearance of PtdIns(4,5)P_2_ (Azzoni et al., 1992; Liao et al., 1992). Lastly, PtdIns(4,5)P_2_ can also be dephosphorylated by the inositol 5-phosphatases INPP5B (Bohdanowicz et al., 2012) and OCRL (Mehta et al., 2014) that are recruited to sites of phagocytosis, producing a transient increase in PtdIns(4)P (Bohdanowicz et al., 2012; Levin et al., 2015).

Breakdown of PtdIns(4,5)P_2_ is required for completion of phagocytosis for two main reasons. Firstly, its removal terminates actin polymerisation at the base of the cup, which is necessary to induce membrane curvature and for the focal secretion of endomembranes. Consistently, inhibition of PLC impairs phagosome formation (Botelho et al., 2000) and is accompanied by persistent actin accumulation at the base of the phagocytic cup (Scott et al., 2005). Secondly, DAG and IP_3_, the two second messengers produced by the PLC-mediated hydrolysis of PtdIns(4,5)P_2_ play important roles in phagocytosis (Bengtsson et al., 1993; Ueyama et al., 2004; Nunes et al., 2012; Schlam et al., 2013). DAG is not only a source of PA following its phosphorylation by DAG-kinase (Bohdanowicz and Grinstein, 2013), but it also activates conventional and novel protein kinase C (PKC) which seemingly participates in phagocytosis (Ueyama et al., 2004) and promotes the activation of the NADPH oxidase (He et al., 2004; Cheng et al., 2007). Furthermore, IP_3_ induces calcium release from the ER that is thought to promote membrane fusion during phagosome formation (Jaconi et al., 1990; Bajno et al., 2000; Braun et al., 2004; Dewitt et al., 2006). Thus, the disappearance of PtdIns(4,5)P_2_ from the base of the phagocytic cup is not purely a consequence of the formation of PtdIns(3,4,5)P_3_, but is in effect, an important outcome of PtdIns(3,4,5)P_3_ formation.

Lastly, accumulation of PtdIns(3,4,5)P_3_, together with the clearance of PtdIns(4,5)P_2_, play an essential role in regulating the ability of receptors to diffuse and recycle by directly removing actin from the base of the phagocytic cup. PtdIns(3,4,5)P_3_ binds and recruits GAPs, such as ARHGAP12, ARHGAP25, and SH3BP1 to the phagocytic cup (Schlam et al., 2015). This, along with the elimination of PtdIns(4,5)P_2_, results in the inactivation of Rho GTPases and prompts the termination of actin polymerisation that would otherwise curtail the mobility of transmembrane proteins (including receptors) via the cytoskeletal picket fence.

3. Phagosome Scission: disappearance of PtdIns(3,4,5)P_3_ and production of PtdIns(3)P

Sealing of the phagosome occurs when the pseudopods fully surround the target particle and fuse at their distal ends. Effectors of PtdIns(3,4,5)P_3_ appear to be crucial for the two main events necessary for proper internalization at this stage. These are (1) clearance of actin surrounding the phagosome (Cox et al., 1999; Beemiller et al., 2010) and (2) constriction of the exofacial leaflets of the plasma membrane where the pseudopods meet to promote scission of the phagosome.

The role of PtdIns(3,4,5)P_3_ and its effectors in mediating actin clearance has been described above. In support of PtdIns(3,4,5)P_3_ being necessary for phagocytic cup formation is the observation that inhibition of PI3K arrests phagosome formation and leads to actin accumulation at the base of frustrated phagosomes (Araki et al., 1996; Cox et al., 1999). Furthermore, expression of constitutively active mutants of Rho GTPases that antagonize actin disassembly yields a similar phenotype of abortive phagocytic cups (Beemiller et al., 2010).

Relatively little is known about the mechanisms behind membrane fusion during scission; however, myosin-driven contractility is probably involved. Myosin X (Cox et al., 2002) and myosin IC (Swanson et al., 1999) localize to sites of phagosome closure, where they are thought to play independent roles during sealing. Interestingly, myosin X harbors a PH domain that binds PtdIns(3,4,5)P_3_ enabling its recruitment to the plasma membrane (Isakoff et al., 1998). Accordingly, the PI3K inhibitor wortmannin prevents myosin X accumulation and expression of a truncated mutant of myosin X reduces phagocytic efficiency (Cox et al., 2002). Surprisingly, the antagonistic effects on phagosomal scission caused by PI3K inhibition seem to be size dependent. PI3K inhibitors only arrest phagocytosis of comparatively large targets (>1 μm) (Cox et al., 2002) while internalization of smaller particles (<1 μm) seems to be largely unaffected (Cox et al., 1999; Vieira et al., 2001).

PtdIns(3,4,5)P_3_ disappears from the phagosomal membrane shortly after scission occurs (Marshall et al., 2001) (Figure 3). Conversion of PtdIns(3,4,5)P_3_ into PtdIns(3,4)P_2_ occurs at this stage since SHIP, a 5-phosphatase (McCrea and De Camilli, 2009), accumulates at the phagosomal membrane (Marshall et al., 2001; Kamen et al., 2007). It is unclear whether PtdIns(3,4)P_2_ plays a role during this stage, other than being a substrate for the formation PtdIns(3)P by INPP4B, a 4-phosphatase (Nigorikawa et al., 2015). PtdIns(3,4,5)P_3_ also can be dephosphorylated by PTEN to regenerate PtdIns(4,5)P_2_ (Maehama and Dixon, 1998).

Lastly, PIP5KIs detach from the membrane of the newly formed phagosome, likely preventing further formation of PtdIns(4,5)P_2_ from PtdIns(4)P (Figure 3). This detachment is partially due to the reduced electronegativity of the early phagosomal membrane, since PIP5KI isoforms contain a polycationic region that preferentially binds negatively charged membranes (Fairn et al., 2009).

## Phagosome Maturation

The maturation of the phagosome is characterized by a series of fission and fusion events that occur soon after sealing. These steps modify both luminal and membrane components of the phagosome and give rise to the ultimate stage of degradation and reabsorption of the cargo during phagosome resolution. The nascent phagosome undergoes stepwise fusion events with early endosomes, late endosomes and lysosomes, which leads to the formation of the early phagosome, late phagosome and phagolysosome, respectively. Microbicidal properties, acidic pH and degradative enzymatic machinery are gradually acquired during phagosome maturation. Nevertheless, it is worth mentioning that differences in the extent and rate of phagosome maturation have been reported between different phagocytic cells (Nordenfelt and Tapper, 2011; Canton et al., 2014). The nature of the engulfed material also accounts for some of the heterogeneity observed during phagosome maturation. For example, phagosomes containing pathogens need to preserve selected peptides for posterior antigen presentation to lymphocytes (Savina and Amigorena, 2007). On the other hand, clearance of apoptotic cells requires rapid acidification and maturation, in addition to secretion of anti-inflammatory cytokines in order to prevent auto-immunity (Ravichandran, 2010; Uderhardt et al., 2012).

As mentioned earlier, phagosome maturation can be additionally sub-classified into three main sequential stages: the early phagosome, the late phagosome and the phagolysosome. These are discussed individually below.

### PtdIns(3)P Defines the Early Phagosome

Phagosome maturation starts as soon as the nascent phagosome detaches from the plasma membrane. Nevertheless, fusion events with endomembranes occur even before phagosome sealing is completed (Bohdanowicz et al., 2012). The newly formed phagosome preferentially fuses with early endosomes (Mayorga et al., 1991; Desjardins et al., 1997), resulting in a poorly degradative, slightly acidic hybrid organelle. Rab family proteins are crucial for vesicular traffic and phagosome fusion events during this and subsequent stages of maturation (Kinchen and Ravichandran, 2008; Fairn and Grinstein, 2012). Rab5 is involved in the early steps and is the prototypical marker of early phagosomes (Bucci et al., 1992; Roberts et al., 2000; Vieira et al., 2003).

Relevant to this review is the fact that the class III phosphatidylinositol 3-kinase, Vps34, is one of the key effectors of Rab5 (Christoforidis et al., 1999; Vieira et al., 2001; Munksgaard et al., 2002). Vps34 is present in early endosomes where it generates PtdIns(3)P by phosphorylating PtdIns on the D3 position. PtdIns(3)P is also the defining phosphoinositide of the early phagosome (Vieira et al., 2001) (Figure 3). Depletion of PtdIns(3)P through pharmacological inhibition of Vps34 arrests phagosome maturation at the early stage (Stephens et al., 1994; Fratti et al., 2001), demonstrating the crucial role of PtdIns(3)P in the progression of phagosomes. Multiple effectors are recruited to the early phagosome by virtue of PX and FYVE domains that bind selectively to PtdIns(3)P.

EEA1 is one of the effectors of PtdIns(3)P that binds the phosphoinositide through its FYVE domain (Simonsen et al., 1998). EEA1 interacts simultaneously with the active form of Rab5 (Mishra et al., 2010) and with PtdIns(3)P in the membrane of early phagosomes and early endosomes. This dual interaction favors early endosome-early phagosome tethering. In addition, EEA1 interacts with the soluble NSF-attachment protein receptors (SNAREs) including syntaxins 6 and 13, which further promotes membrane fusion after tethering (Simonsen et al., 1999; Collins et al., 2002). Accordingly, microinjection of neutralizing EEA1 antibodies arrest phagosome maturation (Fratti et al., 2001).

The disappearance of PtdIns(3)P from the phagosomal membrane signals the termination of the early phagosomal stage. Three different mechanisms could account for PtdIns(3)P disappearance: phosphorylation, dephosphorylation, or hydrolysis. The relative contribution of these pathways is currently unknown; however, the enzymes that can catalyze these reactions are known to be present. PtdIns(3)P can be phosphorylated by PIKfyve on its D5 position, yielding PtdIns(3,5)P_2_ (Burd and Emr, 1998). Additionally, MTM1, a member of the myotubularin family of 3-phosphatases capable of breaking down PtdIns(3)P into PtdIns can also displace Vps34 from endosomal membrane, favoring PtdIns(3)P depletion (Yan and Backer, 2007). Lastly, lysosomal phospholipases can break down PtdIns(3)P (Ching et al., 1999), an event likely to occur upon formation of intraluminal vesicles (ILVs).

Despite the fact that multiple fusion events take place during phagosome maturation, the surface area of the phagosome remains virtually unchanged. This suggests that membrane recycling mechanisms must occur concomitantly. Retrograde transport of phagosomal components to the *trans-*Golgi network is partially responsible for membrane recycling, a process mediated by the retromer complex (Hierro et al., 2007). The retromer is composed of a sorting nexin (SNX) dimer (SNX1/SNX2 and SNX5/SNX6) and a cargo-recognition trimer (Vps26-Vps29-Vps35). SNXs contain a PX domain that serves to recruit them to the early phagosome, where PtdIns(3)P is present. Tellingly, the last steps of retrograde traffic are completed during the late stages of phagosome maturation (Bonifacino and Hurley, 2008).

As previously mentioned, in addition to outward vesiculation, the phagosomal membrane experiences inward budding and generates ILVs destined for degradation (Lee et al., 2005). ILV formation in phagosomes and endosomes is dependent on the endosomal sorting complex required for transport (ESCRT) (Vieira et al., 2004; Babst, 2011). The ESCRT super-complex consists of four smaller complexes (ESCRT-0-III) that together recognize ubiquitinated cargo such as Fcγ receptor (Booth et al., 2002; Wollert and Hurley, 2010). Most relevant to this review, ESCRT-0 bears a FYVE domain-containing subunit, known as Hrs, through which it binds to PtdIns(3)P. Thus, ESCRT-0 gets recruited to the maturing phagosome (Vieira et al., 2004), triggering the assembly of the entire ESCRT super-complex. This results in the inward budding of PtdIns(3)P-enriched ILVs that are degraded at later stages of phagosome maturation.

Reactive oxygen species, a crucial component of the microbicidal properties of phagocytic cells (Nunes et al., 2013), are produced by the action of NOX2 in the phagosomal lumen. Most significant to this review is the fact that p40^phox^, one of the six subunits that make up the oxidase, has a PX domain that binds PtdIns(3)P, making the phosphoinositide crucial for the sustained stimulation of NOX2 in early phagosomes (Ueyama et al., 2007, 2008). Inhibition of PI3K, consequently, prevents the retention of p40^phox^ at the phagosomal membrane and reduces the production of reactive oxygen species (Tian et al., 2008).

### PtdIns(4)P Defines the Late Phagosome

The early phagosome then transitions into a late phagosome, which is more acidic and degradative than earlier stages. A crucial step for this transition is the conversion from a Rab5-positive to a Rab7-positive organelle. As maturation continues, the phagosome migrates toward the microtubule-organizing center (MTOC), which promotes the fusion with late endosomes and lysosomes (Harrison et al., 2003). The active form of Rab7, together with two of its effectors –the Rab7-interacting lysosomal protein (RILP) and the oxysterol-binding protein-related protein 1L (ORP1L)– link the phagosome with the dynein/dynactin motor and are therefore responsible for this centripetal movement (Johansson et al., 2007). Rab7 and RILP also induce the formation of tubular membrane protrusions that promote phagosome-lysosome biogenesis and acidification (Harrison et al., 2003; Sun-Wada et al., 2009).

As mentioned earlier, the retromer is recruited initially to the early phagosome by PtdIns(3)P. Nevertheless, completion of retrograde transport occurs during the late stages through the interaction of Rab7 with the retromer's cargo-recognition trimer (Vps26-Vps29-Vps35). Rab7 depletion affects the structure of the retromer in endosomes and consequently impairs the retrieval of the mannose 6-phosphate receptor to the *trans*-Golgi network (Rojas et al., 2008).

From a phosphoinositide perspective, a marked transition is observed when the early phagosome becomes a late phagosome. Whereas PtdIns(3)P is characteristic of the early phagosome, PtdIns(4)P is the major phosphoinositide present at the late phagosome and phagolysosome stages (Jeschke et al., 2015). Soon after PtdIns(3)P disappears, PtdIns(4)P kinase 2A (PI4K2A), an enzyme responsible for PtdIns(4)P synthesis, accumulates in endosomes (Ketel et al., 2016) and late phagosomes (Jeschke et al., 2015). PtdIns(4)P persists in the phagosomal membrane well into the resolution stage, when its concentration gradually decreases (Figure 3). The accumulation of PtdIns(4)P was shown to be indispensable for proper phagosomal acidification (Levin et al., 2017). A similar role for PI4K2A and PtdIns(4)P as important determinants of maturation has also been recognized in autophagosomes (Albanesi et al., 2015). Yet, the specific effectors of PtdIns(4)P mediating late maturation and resolution are still poorly understood.

### The Phagolysosome

Phagolysosome biogenesis is the next stage in phagosome maturation. Fusion of the late phagosome with lysosomes gives rise to the phagolysosome, the most acidic, degradative and microbicidal organelle. The phagolysosome has an extremely low luminal pH due to the acquisition of additional copies of the proton-pumping V-ATPase. This acidic pH allows for the optimal activity of hydrolytic enzymes (Appelqvist et al., 2013) essential for the ultimate degradation of phagosomal contents.

Of particular interest is the accumulation of PtdIns(3,5)P_2_ in the lysosomal system (Samie et al., 2013; Takatori et al., 2016). PtdIns(3,5)P_2_ is produced via phosphorylation of PtdIns(3)P on its D5 position by PIKfyve (Burd and Emr, 1998). The phosphatase Fig4 harbors a Sac domain and is responsible for the reverse reaction, dephosphorylating PtdIns(3,5)P_2_ back to PtdIns(3)P (Mccartney et al., 2014). PtdIns(3,5)P_2_ breakdown can additionally be catalyzed by myotubularin 3-phosphatases, yielding PtdIns(5)P (Oppelt et al., 2012). Remarkably, PtdIns(3,5)P_2_, PIKfyve and Fig4 all localize to lysosomes, together with the scaffold protein ArPIKfyve/Vac14 (Duex et al., 2006; Jin et al., 2008; Sbrissa et al., 2008). Observations that depleting PtdIns(3,5)P_2_ results in enlarged lysosomes and disrupts lysosomal activity suggest that this complex plays an important functional role in endolysosomes (Ho et al., 2012; Mccartney et al., 2014). Purely in the context of phagocytosis, inhibition of PIKfyve blocks phagosome maturation, seemingly through the inactivation of the transient receptor potential cation channel of the mucolipin subfamily member 1 (TRPML1) (Kim et al., 2014). Previously, PtdIns(3,5)P_2_ was shown to control the activity of TRPML1 (Dong et al., 2010), a cation channel found in lysosomes that promotes Ca^2+^ efflux from the lysosome into the cytosol (Wang et al., 2014). The role of Ca^2+^ in phagosome maturation and endomembrane fusion has been documented (Vergne et al., 2003), accounting for the observation that TRPML1 inhibition blocks fusion of phagosomes and lysosomes (Dayam et al., 2015).

## Phagosome Resolution

The final stage of phagocytosis, phagosome resolution, entails the redirection and degradation of the phagosomal membrane and of luminal components. This stage is also the least well understood. The phagosomal membrane needs to be resorbed once the phagosomal luminal contents are cleared; this includes disposal or recycling of the phosphoinositide constituents of the phagosomal membrane. Earlier stages of phagosome maturation exhibit membrane recycling to the plasma membrane or the *trans*-Golgi network, as well as some degradation through the formation of ILVs. In contrast, relatively little is known about the degradation of the phagolysosomal membrane. In lysosomes, tubulation and fission of vesicles can occur and seems to require the activity of both mTOR and Arl8B (Zoncu et al., 2011; Saric et al., 2016). A similar process putatively promotes the transport of membranous components out of the phagolysosome, analogous to the process observed during antigen presentation (Mantegazza et al., 2014). Evidence from the last decade suggests that PtdIns(4)P and PI4K2A, present in late phagosomes and phagolysosomes, are able to recruit the exocyst, a multimeric complex involved in exocytosis, and mediate membrane recycling to the plasma membrane (Ketel et al., 2016). Further, PtdIns(4)P regulates retromer function and has been linked to actin nucleation via WASH. In the phagolysosomal membrane, WASH and actin-rich regions have also been reported to co-localize with PtdIns(4)P (Levin-Konigsberg et al., 2019) and may serve to propel the initial extension of resorption tubules. In this regard it is interesting to note that PI4K2A and late phagosomal PtdIns(4)P have recently been described to support Toll-like receptor signaling and antigen presentation in dendritic cells (López-Haber et al., 2020).

PtdIns(4)P, which is abundant in maturing phagolysosomes, becomes depleted as the phagolysosome undergoes tubulation and resorbs (Figure 3). PtdIns(4)P can be converted into PtdIns(3,4)P_2_ or PtdIns(4,5)P_2_ by class II PI3Ks (Misawa et al., 1998) and PI5 kinases (Desrivières et al., 1998), respectively. Still, these lipids have not been reliably detected in phagolysosomes and neither have PtdIns(4)P-specific phosphatases. Rather, the main enzyme known to dephosphorylate PtdIns(4)P into PtdIns is Sac1 which resides in the ER (Moser von Filseck et al., 2015). Recently, we showed that PtdIns(4)P is extracted from the phagosomal membrane, and transferred to the ER, where it is available to Sac1 for hydrolysis (Levin-Konigsberg et al., 2019). This removal is facilitated by the lipid transfer protein and Rab7 effector ORP1L at membrane contact sites between the ER and the phagolysosome (Levin-Konigsberg et al., 2019). Furthermore, we showed that tubules emerge from the PtdIns(4)P-rich clusters in the resolving phagolysosome, where ADP-ribosylation factor-like protein 8B (ARL8B) and SifA-and kinesin-interacting protein/pleckstrin homology domain-containing family M member 2 (SKIP/PLEKHM2) accumulate. Accordingly, premature hydrolysis of PtdIns(4)P impairs SKIP recruitment and phagosome resolution (Levin-Konigsberg et al., 2019).

## Concluding Remarks

The development of fluorescent biosensors of PPIns provided an unparalleled tool to investigate the role of these key lipids in leukocyte biology. By enabling their visualization in live cells, we have started to learn about their distribution, dynamics and metabolism under physiologically relevant conditions. These critical determinants of PPIns function could not previously be divined by conventional lipidomic approaches. While great strides have been made in the last two decades using biosensors, it bears emphasizing that the probes are inevitably invasive and that caution must be used limiting their expression, as they can compete for and scavenge endogenous ligands, potentially altering responsiveness. In addition, suitable probes still need to be developed to visualize species like PtdIns(3,5)P_2_, that have been identified as critical determinants of endomembrane traffic and of ion transport. Developing improved probes and applying them to increasingly complex biological systems by intravital and lattice light-sheet microscopy will undoubtedly be the focus of research in the immediate future.

## Author Contributions

All authors listed have made a substantial, direct and intellectual contribution to the work, and approved it for publication.

## Conflict of Interest

The authors declare that the research was conducted in the absence of any commercial or financial relationships that could be construed as a potential conflict of interest.
